# Melanoma-associated fibroblasts decrease tumor cell susceptibility to NK cell-mediated killing through matrix-metalloproteinases secretion

**DOI:** 10.18632/oncotarget.15540

**Published:** 2017-02-20

**Authors:** Linda Ziani, Thouraya Ben Safta-Saadoun, Johanne Gourbeix, Andrea Cavalcanti, Caroline Robert, Gilles Favre, Salem Chouaib, Jerome Thiery

**Affiliations:** ^1^ INSERM, UMR 1186, Villejuif, France; ^2^ Gustave Roussy Cancer Campus, Villejuif, France; ^3^ University Paris Sud, Faculty of Medicine, Le Kremlin Bicêtre, France; ^4^ Department of General Surgery, Gustave Roussy Cancer Campus, Villejuif, France; ^5^ INSERM, UMR 981, Villejuif, France; ^6^ Dermatology Service, Department of Medicine, Gustave Roussy Cancer Campus, Villejuif, France; ^7^ INSERM, UMR 1037, Toulouse, France

**Keywords:** cancer-associated fibroblasts, natural killer cells, matrix-metalloproteinases, MICA/B, melanoma

## Abstract

Cancer-associated fibroblasts (CAFs) play a central role in the complex process of tumor-stroma interaction and promote tumor growth. Emerging evidences also suggest that these fibroblasts are involved in the alteration of the anti-tumor immune response by impacting several immune cell populations, especially through their secretion of pro-inflammatory and immunosuppressive factors in the tumor microenvironment. However, the underlying immuno-modulating mechanisms triggered by these fibroblasts are still only partially defined. In this study, we provide evidence that melanoma-associated fibroblasts decrease the susceptibility of melanoma tumor cells to NK-mediated lysis through the secretion of active matrix metalloproteinases. This secretion reduces the expression of the two NKG2D ligands, MICA/B, at the surface of tumor cells and consequently decreases the NKG2D-dependent cytotoxic activity of NK cells against melanoma tumor cells. Together, our data demonstrate that the modification of tumor cell susceptibility to killer cells is an important determinant of the anti-tumor immune response alteration triggered by CAFs.

## INTRODUCTION

Over the past decade, the tumor microenvironment has gained much attention as a critical determinant of tumor progression and clinical outcome [1–3]. Among the stromal cells, activated fibroblasts that share similarities with fibroblasts stimulated by inflammatory signals or activated during a wound healing process, termed cancer-associated fibroblasts (CAFs), play a critical role in the complex process of tumor-stroma interaction [4, 5]. CAFs, one of the prominent stromal cell population in most types of human carcinomas, are α-SMA (alpha-smooth muscle actin) positive, spindle-shaped cells, who differentiate and proliferate in the tumor microenvironment in a transforming growth factor-β (TGF-β), platelet-derived growth factor (PDGF) and fibroblast growth factor (FGF)-dependent manner from other cell types such as resident fibroblasts, mesenchymal stem cells, endothelial and epithelial cells [5–9]

In the tumor stroma, CAFs play an important role by directly interacting with tumor cells and by the secretion of extracellular matrix proteins (i.e. collagen), matrix metalloproteinases (MMPs), proteoglycans (i.e. laminin), chemokines (i.e. CXCL12/SDF1), vascularisation promoting proteins (i.e. VEGF) and other factors which affect tumor cells proliferation, invasiveness, survival and stemness (i.e. TGF-β) [5, 10–15]. Consequently, CAFs are a key determinant in tumor growth, angiogenesis, cancer stemness, extracellular matrix remodeling, tissue invasion, metastasis and even chemoresistance [5, 16–18].

During the past few years, it has also been either directly demonstrated or suggested that these activated tumor-associated fibroblasts can affect both the innate and the adaptive antitumor immune response, especially by the secretion of pro-inflammatory and immunosuppressive factors in the tumor microenvironment [19, 20]. In this regard, the secretion of TGF-β by CAFs potentially affects several immune cell populations [21], including dendritic cells (by inhibiting their migration, maturation and antigen presentation capabilities [22]), regulatory T cells (Tregs) (by increasing their number within the tumor microenvironment through the induction of FOXP3 expression [23]), cytotoxic T lymphocytes (CTL) (by interfering with their function and frequency within the tumor [24, 25]) and natural killer (NK) cells (by attenuating their interferon-γ (IFN-γ) production and their expression of NK-activating receptors including NKG2D, NKp30 and NKp44 [26–28]). Similarly, CAFs are a source of VEGF, which is known to affect dendritic cell function, to inhibit the migration of CTL to the tumor and to increase the infiltration of Tregs and immunosuppressive myeloid-derived suppressive cells (MDSC) within the tumor [29]. Moreover, the secretion of several chemokines by CAFs (including CXCL12/SDF1 and CCL2/MCP-1) can potentially attract macrophages in the tumor microenvironment and induce their differentiation into an M2 immunosuppressive phenotype [30, 31], and also allow the recruitment of immunosuppressive MDSC populations in the stroma [32, 33]. Finally, studies involving melanoma, hepatocellular and colorectal carcinoma-derived fibroblasts have shown that CAFs can decrease the expression of several NK activating receptors (including NKp30, NKp44 and NKG2D) on the NK cell surface through the secretion of prostaglandin E2 (PGE2) and/or indoleamine-2,3-dioxygenase (IDO) [34–36] leading to an attenuate cytotoxic activity of NK cells against their tumor target cells.

These findings highlight the action of CAFs on various immune cell populations involved in the antitumor immune response. However, direct evidence supporting that CAFs also interfere with the tumor cell susceptibility to CTL or NK cell-mediated lysis is still lacking. In this study, we found that melanoma-associated fibroblasts decrease the susceptibility of melanoma tumor cells to NK-mediated lysis through the secretion of high levels of active MMPs, which reduce the NKG2D-dependent cytotoxic activity of NK cells by inducing the shedding of two NKG2D ligands, MICA/B, at the surface of tumor cells.

## RESULTS

### Phenotypic characterization of human fibroblasts isolated from melanoma and normal skin

**Figure 1:**
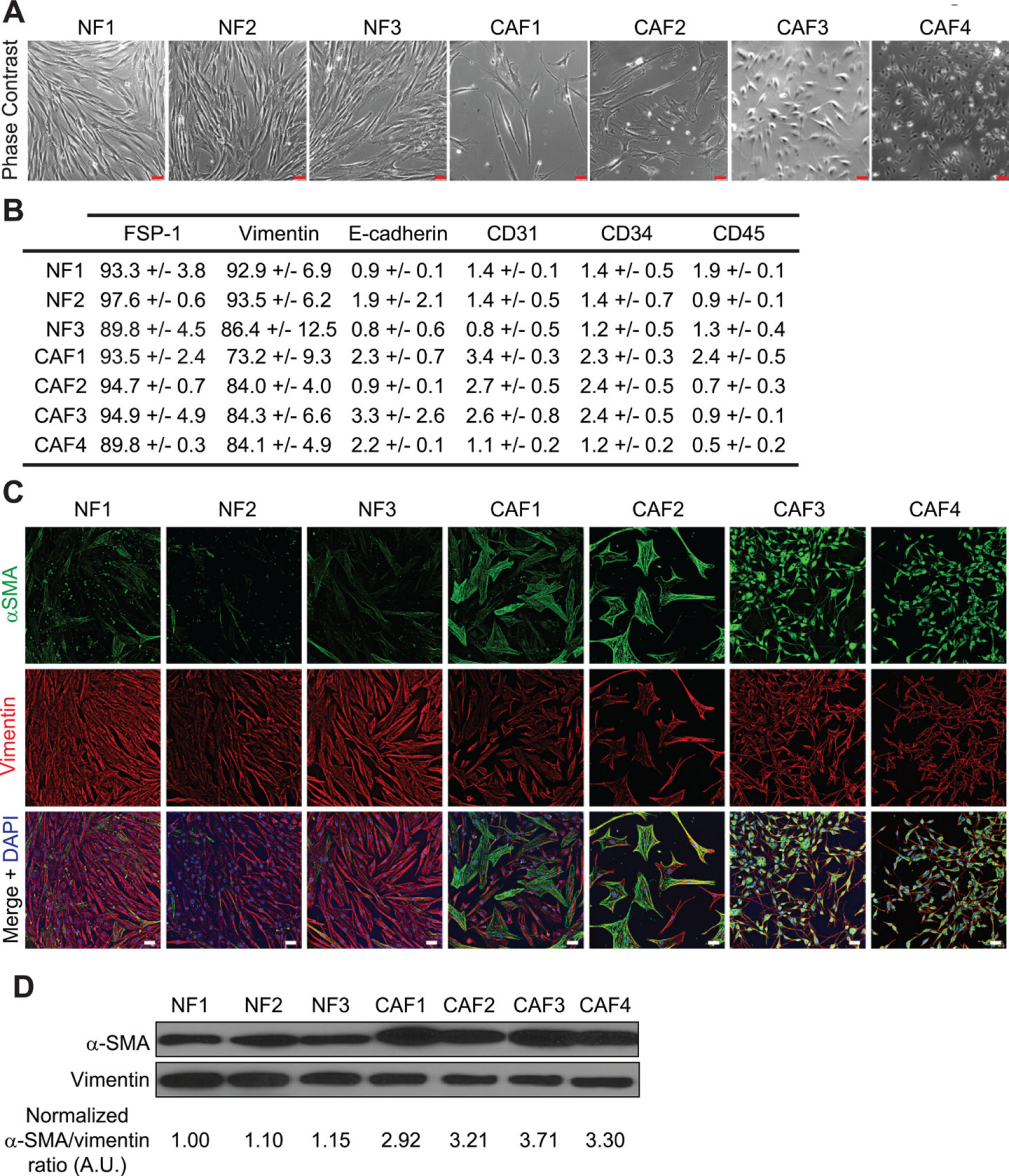
Phenotypes of fibroblasts derived from melanoma and normal skin (**A**) Morphology of the different fibroblastic primary cultures by phase contrast microscopy. (**B**) Expression of the fibroblastic, mesenchymal epithelial, endothelial, hematopoietic or leukocytic markers FSP-1, Vimentin, E-cadherin, CD31, CD34 or CD45 by the different isolated fibroblast populations. Data are expressed as the percentage of positive cells ± s.d. from three independent flow cytometry experiments. (**C**–**D**) CAFs isolated from melanoma patient's tumor biopsies display an activated phenotype. The expression of the activation marker α-SMA, together with the mesenchymal marker Vimentin, was evaluated by fluorescence microscopy (C) and western blot (D). CAFs express more α-SMA than normal skin fibroblasts. The α-SMA/Vimentin ratio from (D) was calculated by densitometry and normalized to “1” in NF1 cells (A.U.: arbitrary units). Data (C–D) are representative of three independent experiments. Scale bars (A, C): 50 μm

In order to study the role of melanoma-associated fibroblasts on the modulation of melanoma tumor cell susceptibility to NK cell-mediated lysis, we established four CAFs and three normal skin fibroblasts (NF) primary cell populations from melanoma patient's tumor resection and from normal skin (Table 1). The adherent cells, which display a fibroblast-like morphology (i.e. elongated cells with cytoplasmic extensions) (Figure 1A) were further characterized by flow cytometry and fluorescence microscopy. Both melanoma and normal skin-derived cell populations express the fibroblastic marker FSP-1 (Fibroblast Specific Protein-1) and the mesenchymal marker vimentin, but not the epithelial marker E-cadherin, the endothelial marker CD31, the hematopoietic marker CD34 or the leukocyte marker CD45 (Figure 1B). Moreover, the melanoma-derived fibroblasts (CAF1-4) exhibit an activated phenotype as shown by a higher α-SMA expression compared to the fibroblasts derived from normal skin (NF1-3) (Figure 1C–1D). These observations indicate that these cell populations are mainly fibroblasts, with minimal contamination with epithelial, endothelial or hematopoietic cells, and confirm the previously described different activation level between tumor-derived and normal fibroblasts.

**Table 1:** List of the different fibroblast populations

Primary fibroblast culture	Origin	Stade
NF1	Normal skin	-
NF2	Normal skin	-
NF3	Normal skin	-
CAF1	Melanoma	Metastatic
CAF2	Melanoma	Metastatic
CAF3	Melanoma	Metastatic
CAF4	Melanoma	Primitive

NF: normal fibroblast. CAF: cancer-associated fibroblast.

### Melanoma-associated fibroblasts decrease tumor cell susceptibility to NK-mediated lysis

**Figure 2:**
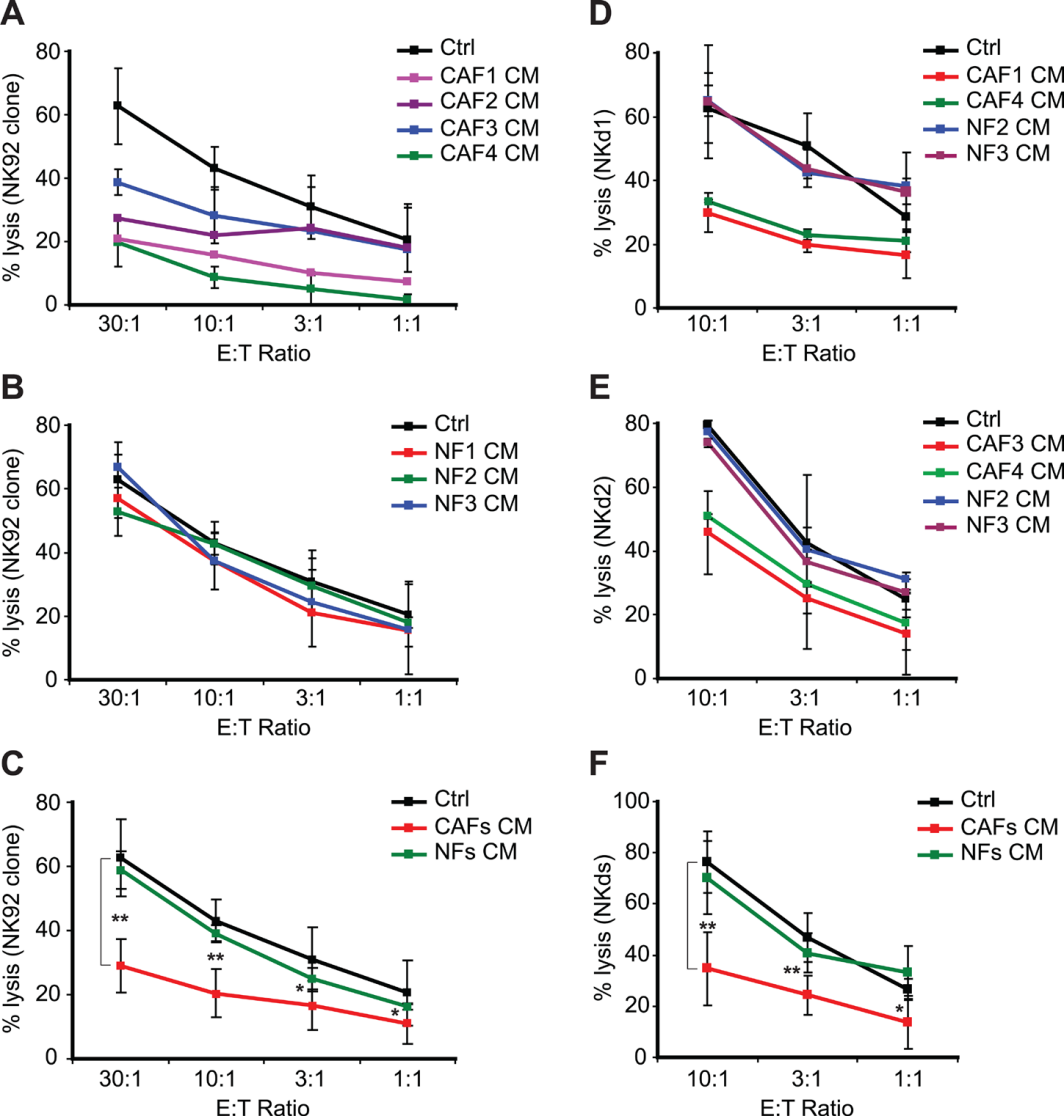
Conditioned media from melanoma-associated fibroblasts decrease the susceptibility of tumor cells to NK-mediated killing (**A**–**C**) The lysis of the T1 melanoma cell line, untreated (Ctrl) or pre-treated during 48 hrs with the conditioned media (CM) of CAFs (CAF1-4) (A) or normal skin fibroblasts (NF1-3) (B) by the NK92 clone was evaluated by ^51^Cr release assays at different effector:target (E:T) ratios. Data are the mean ± s.d. from five independent experiments. Experiments in (A–B) were performed at the same time but separated in two different panels. (C) represents the mean ± s.d. of all the NK92-mediated lysis experiments from (A–B) using the CAFs or NFs pre-treatments of the T1 tumor cell line. (**D**–**E**) The lysis of the T1 melanoma cell line, untreated (Ctrl) or pre-treated during 48 hrs with the conditioned media (CM) of CAFs (CAF1, 3 or 4) or normal skin fibroblasts (NF2-3) by the NK cells isolated from two healthy donors (NKd1 and NKd2) was evaluated by ^51^Cr release assays at different effector:target (E:T) ratios (D–E). Data are the mean ± s.d. from three independent experiments. (**F**) represents the mean ± s.d. of all the NKd-mediated lysis experiments from (D–E) using the CAFs or NFs pre-treatments of the T1 tumor cell line. *P* values (C–D) were determined by unpaired two-tailed student's *t-test* comparing the control and CAFs CM pre-treatments. (*p < 0.05; **p < 0.001)

The pro-tumorigenic activity of CAFs includes direct cellular interactions and mostly paracrine effects affecting different cell types present in the tumor microenvironment. To investigate the effect of melanoma-derived CAFs on melanoma tumor cell susceptibility to NK cell-dependent killing, focusing on their “secretome”, we generated conditioned medium (CM) from the culture supernatants of melanoma-derived fibroblasts (CAF1-4) and normal skin-derived fibroblasts (NF1-3) and used them to treat a melanoma tumor cell line establish from a tumor biopsy (T1) during 48 hrs. The lysis of T1 target cells by the NK92 clone was thus strongly decreased after pre-treatment with the CAF1-4 CMs while NF1-3 CMs have no effect (Figure 2A–2C). To confirm these results, we used NK cells isolated from two different healthy donor's peripheral blood (NKd1 and NKd2) to test their lytic potential against the T1 melanoma target cells pre-treated with the CAF1,3,4 and NF2,3 CMs. As expected, the NKd1 or NKd2-mediated lysis of T1 target cells was also strongly decreased after pre-treatment with the CAF1-4 CMs while NF2-3 CMs have no effect (Figure 2D–2F). Similar results were also obtained using the melanoma tumor cell line WM17-16 and NKd1 effector cells (Supplementary Figure 1).

We then tested whether CAF CMs affect NK cells adhesion to T1 target cells by measuring the immune conjugate formation between T1 cells and NK92 effector cells. CAF or NF CMs-pretreated (48 hrs) or control T1 target cells and NK92 were respectively stained with the lypophilic dyes DiO or DiD and conjugates formation was measured by flow cytometry after 30 min of co-culture. No significant differences were observed for the formation of immune conjugates between NK92 cells and T1 control cells or T1 target cells pretreated with either the CAFs or the NFs CMs (Supplementary Figure 2A–2B). To further confirm these results, we also evaluated ICAM-1/CD54 expression at the surface of T1 targets cells, since its interaction with LFA-1 contributes to NK cells adhesion to targets cells. Consistently with the lack of difference in the formation of immune conjugates between NK92 cells and T1 control cells or T1 target cells pretreated with either the CAFs or the NFs CMs, ICAM-1 surface expression was similar in either control or CMs-treated T1 cells (Supplementary Figure 2C). Because the lysis of the T1 tumor target cells by the NK92 clone and by NK cells isolated from healthy donor's is mainly mediated by the Perforin/Granzymes (PFN/Gzms) pathway, as shown by abrogation of NK92 and NKds cytotoxicity after treatment with concanamycin A (CMA) which inhibits cytotoxic granules exocytosis (Supplementary Figure 3A), we also tested whether the CAFs or the NFs CMs alter T1 tumor cell susceptibility to PFN/Granzyme B (GzmB)-induced cell death by measuring the activation of effector caspases in either control or CMs-pre-treated cells. We used a flow cytometry-based assay using M30-FITC mAbs to detect a caspase-3 cleavage product of cytokeratin 18 (CK18) [37, 38]. Again, no significant differences were observed for PFN/GzmB-induced apoptosis between T1 control cells or T1 cells pre-treated with either the CAFs or the NFs CMs (Supplementary Figure 3B). Together, these results indicate that melanoma-associated fibroblasts protect melanoma tumor cells against NK-mediated cytotoxicity by a mechanism which is not associated with an alteration of tumor cell recognition or with a decrease of tumor cell susceptibility to PFN/GzmB-induced cell death.

### Melanoma-associated fibroblasts decrease MICA/B expression on tumor cells

**Figure 3:**
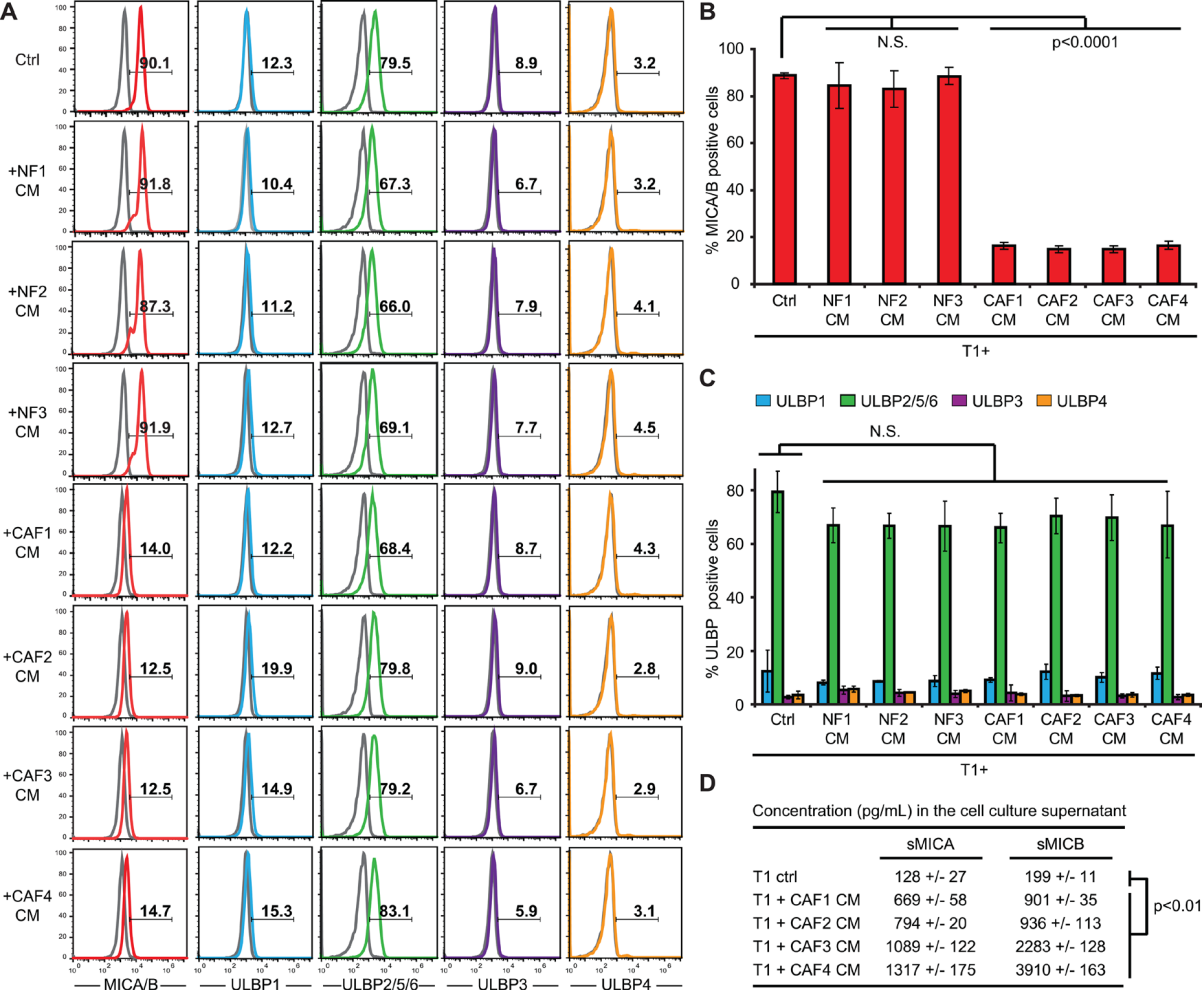
Conditioned media from melanoma-associated fibroblasts decrease MICA/B expression at the surface of tumor cells (**A**–**C**) The expression of MICA/B, ULBP1, ULBP2/5/6, ULBP3 and ULBP4 was evaluated at the surface of T1 melanoma cell line, untreated (Ctrl) or pre-treated during 48 hrs with the conditioned media (CM) of CAFs (CAF1-4) or normal skin fibroblasts (NF1-3). Representative flow cytometry histograms (A) and mean ± s.d. of percentage MICA/B (B) and ULPBs (C) positive cells from three independent experiments are shown. Gray lines in A represent the isotype controls. (**D**) The shedding of MICA/B at the surface of T1 tumor cells following a 48 hrs treatment with the conditioned media (CM) of CAFs (CAF1-4) or with control medium, was evaluated by ELISA measuring soluble MICA (sMICA) and MICB (sMICB) concentration (pg/mL) in the cell culture supernatant. Data are represented as mean concentrations ± s.d. from two independent experiments performed in duplicate. *P* values (B-D) were determined by unpaired two-tailed student's *t-test*.

NK cell functions are regulated by a balance of activating and inhibiting signals triggered by membrane receptors expressed by NK cells and their corresponding ligands expressed by target cells [39]. Among these receptors, the activating receptor NKG2D/CD314 is of major importance for NK cell activation and cytotoxic or secretory functions [40]. NKG2D (Natural Killer Group 2 member D) recognizes ligands from the MIC (MHC class I chain-related protein) and ULBP (HCMV UL16-binding proteins) families which appear on the surface of stressed, transformed or infected target cells. In humans, there are currently eight known members of the MIC and ULBP families: MICA, MICB and ULBP 1-6 [40]. In order to determine whether an alteration of the NKG2D/NKG2D ligands activating pathway might be involved in the decreased susceptibility of melanoma tumor cells to NK-mediated lysis following CAFs CMs treatment, we first determined whether this pathway is involved in NK-mediated killing of the T1 cell line. All NK effector cells used in this study (NK92, NKd1 and NKd2) expressed the NKG2D receptor (Supplementary Figure 4A). Moreover, the use of an anti-NKG2D blocking mAb strongly decreased NK92-, NKd1- and NKd2-mediated killing of T1 melanoma cells (Supplementary Figure 4B), demonstrating that NKG2D is an important determinant for the lysis of T1 cells by NK cells. We then tested the NKG2D ligands expression at the surface of T1 melanoma cells and investigated whether the pre-treatment of these cells with CAFs or NFs CMs can alter their membrane expression. T1 cells strongly express MICA/B and ULBP2/5/6, very slightly express ULBP1 and are negative for ULBP3 and ULBP4 (Figure 3). Importantly, the pre-treatment of T1 cells with the CAFs or NFs CMs does not modify ULBPs surface expression (Figure 3A, 3C), but the pre-treatment of T1 cells with the CAFs CMs strongly decreases MICA/B membrane expression, while the NFs CMs have no effect (Figure 3A–3B). Furthermore, we evaluated by ELISA the presence of soluble MICA (sMICA) or MICB (sMICB) in tumor cell supernatants following treatment with the CAFs CMs. The concentration of both sMICA and sMICB were thus significantly increased in the supernatant of T1 melanoma tumor cells after treatment with the CAF1-4 CMs compared to control cells (Figure 3D). Together, these results suggest that melanoma-associated fibroblasts protect melanoma tumor cells against NK-mediated cytotoxicity by the secretion of soluble factors leading to a decrease of MICA/B expression at the surface of tumor cells, most likely by a mechanism which involved MICA and MICB shedding at the melanoma tumor cell surface.

### The secretion of high levels of active matrix metalloproteinases by melanoma-associated fibroblasts is associated with the decrease of MICA/B expression on tumor cells

**Figure 4:**
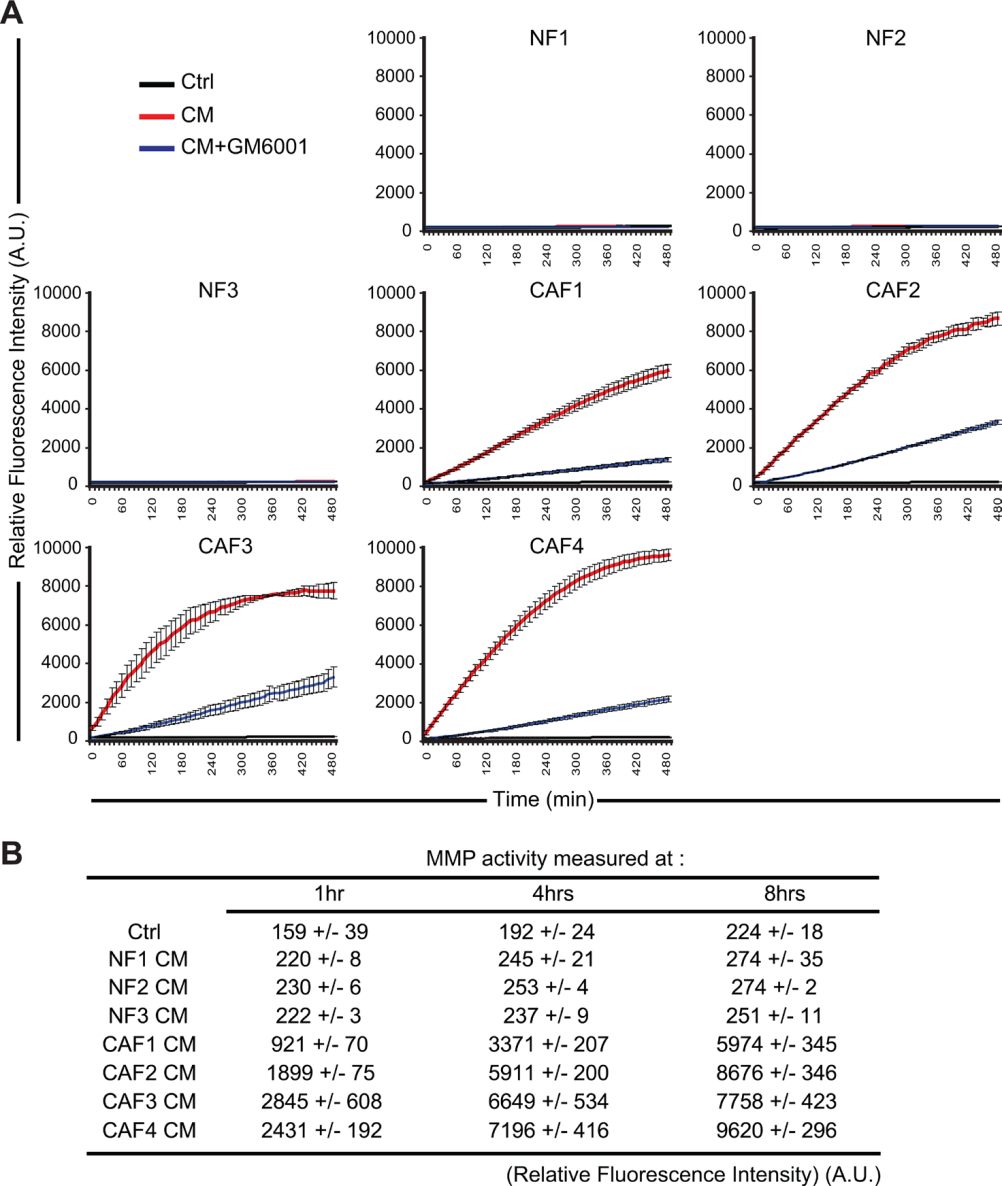
Conditioned media from melanoma-associated fibroblasts contain a high level of active matrix metalloproteinases The activity of MMPs was evaluated in the conditioned media (CM) of CAFs (CAF1-4) or normal skin fibroblasts (NF1-3) in the presence or absence of the MMPs inhibitor GM6001 by measuring the fluorescence emission of a 5-FAM/QXL520 FRET peptide as a MMP substrate. Fluorescence was measured every 10 min. A representative experiment performed in triplicate for each CMs is displayed in (**A**). The relative mean fluorescence intensity ± s.d of two independent experiments, measured at the indicated time (1, 4 and 8 hrs), is represented in (**B**). (A.U.: arbitrary units)

**Figure 5:**
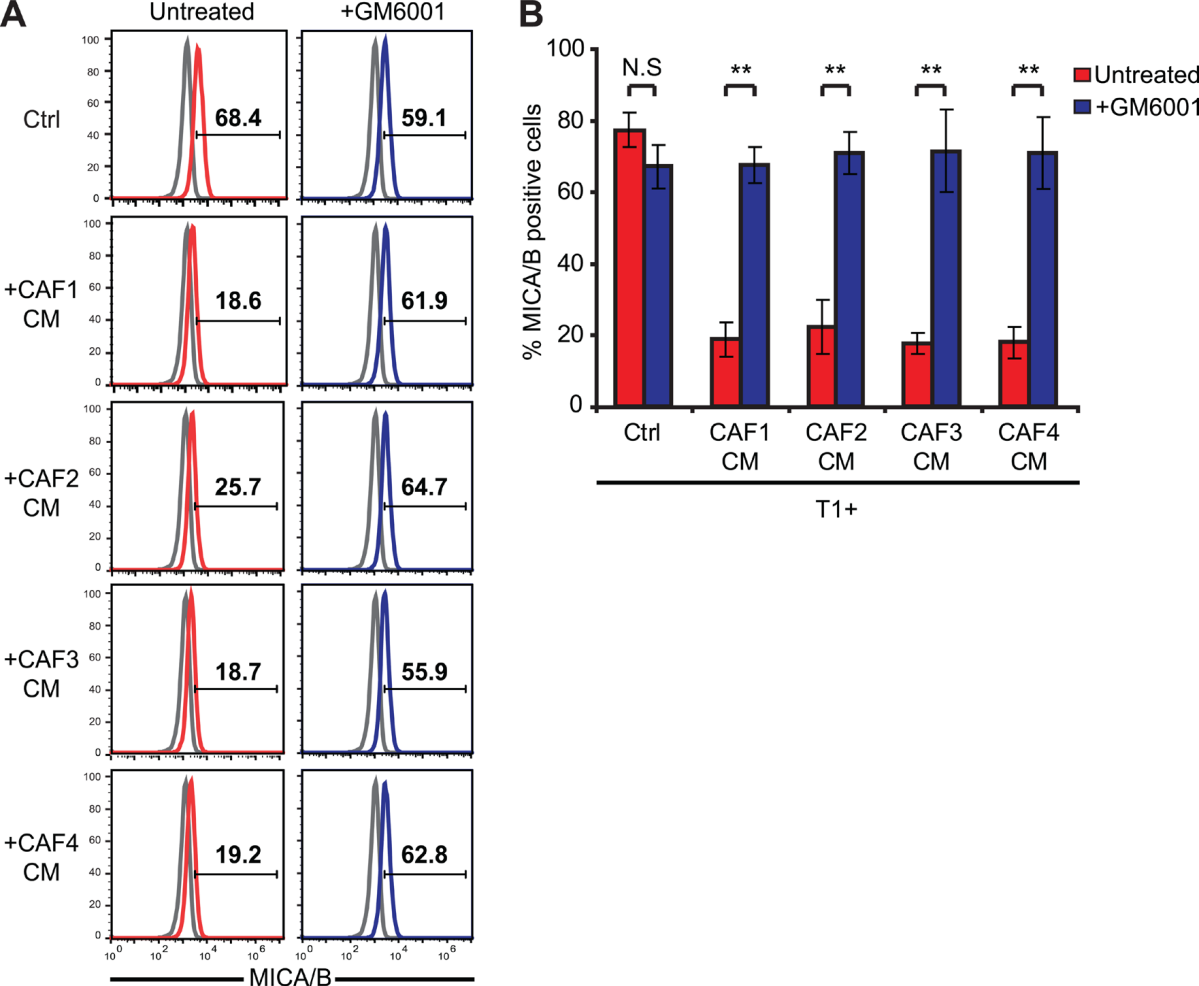
The inhibition of MMPs activity in melanoma-associated fibroblasts conditioned media restores the expression of MICA/B at the surface of tumor cells The expression of MICA/B was evaluated at the surface of T1 melanoma cells, untreated (Ctrl) or pre-treated during 48 hrs with the conditioned media (CM) of CAFs (CAF1-4) in the absence or presence of 50 μM of the MMPs inhibitor GM6001. CAFs CMs or control medium were pre-treated with GM6001 during 15 min before starting the 48 hrs incubation period. Representative flow cytometry histograms (**A**) and mean ± s.d. of percentage MICA/B positive cells (**B**) from three independent experiments are shown. *P* values (B) were determined by unpaired two-tailed student's *t-test*. (N.S: non significant; **p < 10^−5^). Gray lines in A represent the isotype controls.

Although NKG2D ligands, including MICA and MICB, are up-regulated during malignant transformation in response to oncogenic activation, their expression can be down-regulated both transcriptionally and non-transcriptionally at the level of tumor cells to escape antitumor the immune response [41]. For example, the secretion of TGF-β or IFN-γ down-regulates NKG2D ligands transcription in tumor cell lines [42–44]. In addition, several studies have reported that the secretion or expression of MMPs by tumor cells (including the secreted MMP-2, MMP-9 and the membrane type MMP-14) can lead to the shedding of MICA/B at their surface [45–51]. MMPs belong to a group of 26 human zinc-binding endopeptidases which are secreted or anchored to the cell membrane and involved in the degradation of different components of the extracellular matrix [52]. As such MMPs play an important role in tumor cell migration and invasion [53]. Importantly, CAFs also play an important role in the modification of the extracellular matrix by expressing a large variety of matrix remodeling enzymes such as MMPs [16]. We thus hypothesized that high levels of active MMPs secreted by our melanoma-associated fibroblasts could induce the observed decrease of MICA/B and shedding at the surface of the T1 melanoma tumor cells. To validate this hypothesis, we first evaluate MMPs activity in the CAFs and NFs CMs. We used a fluorescence-based assay to measure the activity of MMP-1, 2, 3, 7, 8, 9, 12, 13, and 14. This assay is based on a 5-FAM/QXL520 fluorescence resonance energy transfer (FRET) peptide as an MMP substrate. In the intact FRET peptide, the fluorescence of 5-FAM is quenched by QXL520. Upon cleavage into two separate fragments by MMPs, the fluorescence of 5-FAM is recovered and can be monitored. Using this approach, we showed that CAFs CMs contain higher levels of active MMPs as compared to the CMs of NFs. Moreover, the MMPs inhibitor GM6001 (Galardin/Ilomastat) which inhibits the activity of secreted MMP-1, 2, 3, 7, 8, 9 and 12 strongly inhibits the MMPs activity in these CAFs CMs (Figure 4A–4B). To validate the implication of MMPs in the decreased membrane expression of MICA/B on T1 tumor cells following treatment with the CAFs CMs, CAFs CMs were then pre-treated with 50 μM GM6001 during 15 min before incubating T1 tumor cells with these CMs during 48 hrs. Flow cytometry analysis revealed that GM6001 prevents the decrease of MICA/B surface expression induced by the CAFs CMs (Figure 5A–5B).

Together, these results indicate that melanoma-associated fibroblasts secrete high levels of active MMPs leading to a decrease of MICA/B expression at the membrane of tumor cells.

### The inhibition of matrix metalloproteinases activity in melanoma-associated fibroblasts conditioned media restores tumor cell susceptibility to NK-mediated lysis

**Figure 6:**
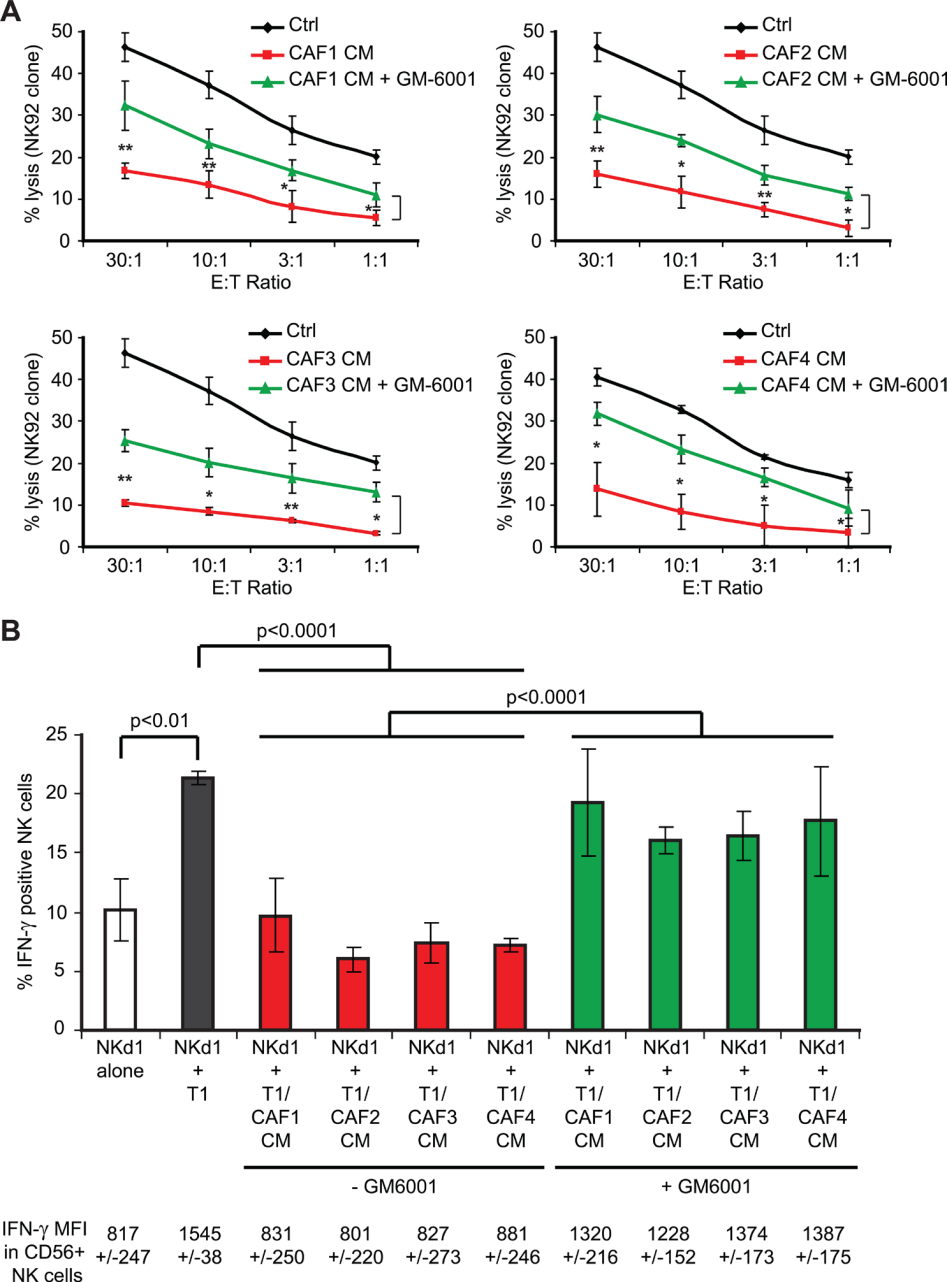
The inhibition of MMPs activity in melanoma-associated fibroblasts conditioned media partially restores tumor cell susceptibility to NK cell-mediated lysis and interferon-g production by NK cells (**A**) NK92-mediated lysis of the T1 melanoma cell line, untreated (Ctrl) or pre-treated during 48 hrs with the conditioned media (CM) of CAFs (CAF1-4) in the presence or absence of the MMPs inhibitor GM6001 was evaluated by ^51^Cr release assays at different effector:target (E:T) ratios. (**B**) The IFN-γ production by NK cells isolated from a healthy donor (NKd1) was measured by flow cytometry after 6 hrs co-culture with T1 tumor cells, either untreated (Ctrl) or pre-treated during 48 hrs with the conditioned media (CM) of CAFs (CAF1-4) in the presence or absence of the MMPs inhibitor GM6001.Co-cultures were performed in the presence of Brefedin-A to block the IFN-γ secretion before CD56 and IFN-γ staining. The percentage of IFN-γ positive NK cells is represented and the mean fluorescence intensity (MFI) of IFN-γ in CD56 positive NK cells is indicated below. Data (A–B) are the mean ± s.d. from three independent experiments. *P* values were determined by unpaired two-tailed student's *t-test*. (*p < 0.05; **p < 0.01)

In order to validate the implication of MMPs in the decreased susceptibility of T1 tumor cell susceptibility to NK cell-mediated lysis following treatment with the CAFs CMs, we next tested whether the restoration of MICA/B expression in the presence of the MMPs inhibitor GM6001 can increase CAFs CMs-treated tumor cell-susceptibility to NK cell-mediated killing. For this purpose, T1 tumor cells were treated with control medium or CAFs CMs in the presence or absence of 50 μM GM6001 during 48 hrs before measuring NK92 cell cytotoxic activity. As previously described, the treatment of T1 tumor cells with CAF1-4 CMs decreases their susceptibility to NK92-mediated lysis. However, the presence of the MMPs inhibitor GM6001 in the CAF1-4 CMs partially restores (~50%) the killing of T1 tumor cells by the NK92 effector cells (Figure 6A). To further validate these results, and since NKG2D engagement by MICA/B not only triggers the cytolytic activity of NK cells, but also their cytokine production, we next evaluated IFN-γ production by NK cells isolated from a healthy donor (Nkd1) co-cultivated with T1 melanoma target cells, pre-treated with CAFs CMs or control medium, in the presence or absence of GM6001. Similarly to NK-mediated lysis, the treatment of T1 tumor cells with CAF1-4 CMs decreases the induction of IFN-γ production by NKd1 cells after 6 hrs of co-culture (while NF1-3 CMs have no effect; Figure 6B and data not shown). Moreover, the presence of the MMPs inhibitor GM6001 partially restores the IFN-γ production (~70%) of NKd1 cells following incubation with the CAF1-4 CMs-treated T1 target cells (Figure 6B). Together, these results correlate with the restoration of MICA/B expression at the surface of CAFs CMs-treated T1 tumor cells following GM6001 treatment and demonstrate that MMPs present in the CAFs CMs are, at least partially, responsible for the decreased susceptibility of melanoma tumor cell susceptibility to NK cell-mediated lysis though a diminished NKG2D-dependent NK cell activation.

## DISCUSSION

NK cells can participate to the early immune response against melanoma and contribute to the adaptive immune response by the secretion of cytokines and by the promotion of antigen-presenting cell maturation. However, even if melanoma cells are often efficiently recognized and killed by NK cells *in vitro*, melanomas have developed multiple elaborated strategies to escape from NK cell-mediated destruction *in vivo* [54, 55]. In the present study, we provide evidence that fibroblasts isolated from melanoma tumor biopsies strongly interfere with melanoma tumor cell susceptibility to NK-mediated lysis. This suppressive effect is largely dependent on high levels of active MMPs released by melanoma-associated fibroblasts and is specific of the fibroblasts present in the tumor microenvironment because normal skin fibroblasts only minimally affect melanoma tumor cell susceptibility to NK-mediated killing.

It is now well established that different soluble factors released by tumor cells in the tumor microenvironment (i.e. TGF-β, PDGF and FGF) locally activate fibroblasts which acquire phenotypic and functional properties different from their normal counterparts [5]. In particular, when exposed to these stimuli, CAFs express proteases such as MMPs, which can favor remodeling of the extracellular matrix (ECM) and promote tumor invasion. Indeed, normal fibroblasts continually regulate and restrain the change of the ECM in healthy tissue by strictly controlling MMPs secretion and membrane-type matrix metalloproteinases (MT-MMPs) expression, thereby preventing the degradation of basement membrane and consequently blocking a potential metastasis process. On the opposite, soluble factors present in the tumor microenvironment such as TGF-β, TNF-α, IL1β and FGF promote the expression of MMPs by fibroblasts [56, 57]. Accordingly, we observed in our study that melanoma-associated fibroblasts secrete high level of active MMPs while normal skin fibroblasts only display minor MMPs activity in their CMs. Importantly, this high level of MMPs activity in the CMs of melanoma-derived fibroblasts is associated with the decreased susceptibility of melanoma tumor cells to NK-mediated lysis, as shown by the use of the pan-MMPs inhibitor GM6001 which restores melanoma tumor cells susceptibility to NK-mediated killing in the presence of melanoma-associated fibroblasts CMs. Of note, the inhibition of MMPs activity in the CAFs CMs using GM6001 is highly effective (Figure 4) but only partially restores melanoma tumor cell susceptibility to NK-mediated attack (Figure 6A), suggesting that MMPs may not be the sole factor involved.

With regard to the mechanism of inhibition, our data provide evidence that melanoma-associated fibroblasts, though the secretions of active MMPs, decrease MICA/B expression at the surface of melanoma tumor cells. In our model, this effect of melanoma-derived CAFs seems to be specific to these two NKG2D ligands because ULPBs expression is not affected, even if we cannot exclude that the secretion of active MMPs or other factors by CAFs might also influence the expression of other activating receptor ligands (i.e. CD112, CD155, B7-H6, HLA-E) which respectively bind to DNAM-1, NKp30 and NKG2C. Nevertheless, because the activating receptor NKG2D/NKG2DL pathway is of major importance for NK activation, cytolytic functions and cytokine secretion, the observed CAFs and MMPs-dependent decrease in MICA/B expression at the surface of melanoma tumor cells leads to a strong alteration of the NK cells cytotoxic activity against their melanoma target cells, as well as a diminished secretion of IFN-γ by NK cells following recognition of their targets. Remarkably, this phenomenon seems to be conserved, as all the CAFs isolated from four different melanoma patient's tumor resection have the same effect on NK cell-dependent killing. Nevertheless, further studies will be needed to determine whether this phenomenon might be extended to CAFs from other tumor tissues. Of note, since NKG2D on some CD8^+^ T cells can trigger a co-stimulatory signal [58, 59], CAF-dependent decrease of MICA/B expression at the surface of melanoma tumor cells might also affect the T cell-dependent immune response.

Regarding the mechanism of action of MMPs on MICA/B, a few studies have reported that the secretion or expression of these metalloproteinases by tumor cells (including the secreted MMP-2, MMP-9 and MT-MMP-14) can lead to the proteolytic cleavage/shedding of MICA/B [45–51] and possibly ULBP2 and ULBP3 [60] at their surface. Based on our results, it is likely that not only tumor cells can affect MICA/B membrane expression by the secretion of MMPs, but also stromal cells including CAFs. However, further studies will be needed to identify the exact MMPs involved in this CAF-dependent process. Nevertheless, because MT-MMP-14 is normally anchored to the plasma membrane by a trans-membrane and intra-cytoplasmic domain, it is unlikely that this MMP is, at least directly, involved in the shedding of MICA/B in our study which uses CMs. Moreover, GM6001 mostly inhibits the activity of secreted MMP-1, 2, 3, 7, 8, 9 and 12 and almost completely prevents the shedding of MICA/B at the surface of tumor cells treated with the CAFs CMs (Figure 5), suggesting that one or several of these MMPs are involved. Moreover, RT-qPCR experiments showed that CAF1-4 mostly expressed MMP1,2,3 and 9 at the mRNA level (data not shown), narrowing the list of MMPs possibly involved in the CAF CMs-dependent shedding of MICA/B at the surface of melanoma tumor cells to MMP1,2,3 and 9.

Finally, it is important to point out that a pioneer study involving CAFs isolated from melanoma, but also other studies involving hepatocellular and colorectal carcinoma-derived fibroblasts, have shown that these cells can decrease NKp30, NKp44, DNAM-1 and/or NKG2D expression at the surface of NK cells, as well as PFN or GzmB expression [34–36]. These effects seem to be dependent, at least partially, on the secretion of PGE2 and IDO by CAFs and lead to an attenuate cytotoxic activity of NK cells against their tumor target cells. In this context, these studies and our results highlight that the CAFs secretome, especially in melanoma, profoundly alter the NK-dependent antitumor immune response by different mechanisms, including the secretion of active MMPs. Further studies will be thus required to determine whether the use of MMPs inhibitors [61] might improve the NK-dependent control of tumor growth in preclinical models, or might be helpful in combination with the current immune-based anti-tumor/anti-melanoma therapies.

## MATERIALS AND METHODS

### Cell lines

T1 and WM17-16 melanoma tumor cells were grown in RPMI-1640/Glutamax™ supplemented with 10% FCS, 100 U/ml penicillin, 100 mg/ml streptomycin and 1% sodium pyruvate (Life Technologies). The T1 tumor cell line was established from the primary lesion of a patient suffering from a melanoma [62]. The metastatic melanoma cells WM17-16 were obtained from ATCC. The NK92 cell line and NK cells isolated from healthy donors (NKd) with human CD56 positive selection kit (StemCell) were cultured in RPMI 1640/GlutaMax™ supplemented with 10% of FCS and 300 U/mL rhIL2 (Sanofi).

### Isolation of melanoma-associated and normal skin fibroblasts

A panel of 4 pathology-confirmed melanoma tumor resections and 3 normal skin biopsies from healthy donors was obtained in accordance with consent procedures approved by the Gustave Roussy Institute. A mechanical dissociation by cutting and scrapping the tissue into small pieces (1–2 mm^3^) was performed in a 10 cm culture plate. The pieces were then distributed into a 24 wells plate and fibroblast growth medium (DMEM-F12, 10% FCS, 100 U/ml penicillin and 100 mg/ml streptomycin) was then added. After several days, outgrowth of tumor-derived cells was observed. Tissue debris and non-adherent cells were then removed and medium changed. After 5–7 days, cells were trypsinized 2–3 min with 0.25% trypsin-EDTA until the fibroblast population was released from the plate as visually assessed by microscopy, whereas tumor cells were retained. The cell suspension was then centrifuged and fresh culture medium was added before the transfer into new wells. Because fibroblasts are highly adherents to plastic, the medium was changed after 30 min to allow the removal of the remaining tumor cells. Cells population homogeneously displaying a fibroblast-like morphology were then phenotypically characterized and assessed for purity by the analysis of the indicated informative markers.

### Preparation of fibroblast-derived conditioned media and treatment of the melanoma tumor cells

CAFs or NFs were seeded at equal density (2 × 10^5^ cells/well in 12 well plates) and maintained in culture during 48 hrs in DMEM/F12 medium supplemented with 5% FCS. Culture supernatants were then collected and centrifuged 15 min at 2,000 rpm to remove cell debris. These supernatants, considered as conditioned media (CM), were then immediately used or frozen at −20°C for subsequent use. The treatment of T1 or WM17-16 tumor cells with the CMs was performed during 48hrs.

### Flow cytometry analysis

Phenotypic analyses of tumor cells, NK cells or fibroblasts were performed by direct or indirect immuno-staining. Briefly, 0.2 × 10^6^ cells were stained with the following Abs: rabbit anti-FSP-1/S100A4 mAb (clone EPR2761, Abcam); rabbit anti-vimentin mAb (clone SP20, Abcam); PE-conjugated mouse anti-human E-Cadherin/CD324 mAb (clone 67A4, Biolegend); Alexa Fluor^®^ 488-conjugated mouse anti-human CD31 (clone M89D3, BD Pharmingen); FITC-conjugated mouse anti-CD34 mAb (clone AC136, Miltenyi Biotec.); PE-conjugated mouse anti-human CD45RO mAb (clone UCHL1, DakoCytomation); PE-conjugated mouse anti-MICA/B mAb (clone 6D4; Biolegend); PE-conjugated mouse anti-human ULBP-1 mAb (clone 170818); APC-conjugated mouse anti-human ULBP-2/5/6 mAb (clone 165903); PE-conjugated mouse anti-human ULBP-3 mAb (clone 166510); PE-conjugated mouse anti-human ULBP-4 mAb (clone 709116) (R&D systems); mouse anti-ICAM-1/CD54(clone 25D7); PE-conjugated mouse anti-NKG2D mAb (clone ON72, Beckman Coulter); Alexa Fluor^®^ 488-conjugated goat anti-rabbit Ab (Life Technologies). For intracellular staining, cells were fixed and permeabilized with CytoFix/CytoPerm solution (BD Biosciences). Extracellular staining were performed at 4°C. Acquisitions were performed using a BD Accuri™ C6 flow cytometer (BD Biosciences) and data were processed using the FlowJo program.

### Fluorescence microscopy

Cells were grown on rat collagen-coated glass coverslips (Sigma) and fixed for 20 min in PBS/2% PFA, washed and incubated 20 min in PBS/50 mM NH4Cl. Cells were then washed with PBS, permeabilized for 5 min in PBS/0.2% Triton X-100. After 2 washes in PBS, coverslips were placed in blocking solution (PBS/10% FCS) for 30 min, washed once in PBS and incubated for 1 hr at RT with mouse anti-α-SMA mAb (clone 4A4, Abcam) and rabbit anti-Vimentin mAb (clone SP20, Abcam) in incubation buffer (PBS/0.05% Triton X-100). Cells were then washed 3 times with incubation buffer and incubated 1 hr at RT with goat Alexa-Fluor 488 and Alexa-Fluor 647-conjugated secondary antibody (Life Technologies) in incubation buffer containing 5% FCS. Cells were then washed 3 times in PBS and mounted in Vectashield mounting medium (Vector Laboratories) before imaging (IX83 microscope; Olympus) and analysis (CellSense Dimension software, Olympus).

### Western blot

Total cellular extracts were prepared by lysing cells in RIPA buffer (Pierce) containing a cocktail of protease inhibitors (Roche) and 2 mM sodium orthovanadate before denaturation by boiling in Laemmli buffer and SDS-PAGE separation on 4–20% precast gels (Biorad). Blots were probed with the following Ab: anti-α-SMA mouse mAb (clone 4A4, Abcam), anti-Vimentin rabbit mAb (clone SP20, Abcam), HRP-conjugated anti-actin mouse mAb (clone AC-74, Sigma), HRP-conjugated goat anti-mouse or anti-rabbit Ab (Santa Cruz Bio.). Western blot quantification was performed using the Image-J densitometry software.

### Chromium release assay

Cytotoxicity was measured by a 4 hr chromium release assay as previously described [63]. Experiments were performed in triplicate. Data are expressed as the percentage of specific ^51^Cr release from target cells, calculated as (experimental release-spontaneous release)/(maximum release-spontaneous release) × 100. In some experiments, the inhibition of the PFN/Gzm-mediated cytotoxic pathway was performed by using NK effector cells pre-incubation for 2 hr with 100 nM concanamycin A (CMA)(Sigma-Aldrich) (concentration of CMA during lysis: 50 nM). The inhibition of the NKG2D-dependent cytotoxic was performed by using NK effector cells pre-incubation for 30 min with 0.5 μg/mL anti-NKG2D blocking mAb (clone Bat-221; Miltenyi Biotec.) which was maintained at the same concentration during lysis.

### Measurement and inhibition of MMPs activity in conditioned media

The metalloproteinases activity in CAFs and NFs CMs was measured using the fluorometric SensoLyte^®^ 520 Generic MMP Activity Kit (AnaSpec) according to a modified manufacturer protocol. Briefly, the supernatants of CAFs or NFs (cultured during 48 hrs without FCS) were collected and centrifuged for 10–15 min at 1,000 g at 4°C. MMPs substrate and MMPs diluents were then mixed together (50 μL) in a 96 wells plate before adding 50 μL of the freshly prepared CAFs or NFs CMs to initiate the enzymatic reaction. Fluorescence was then immediately measured and monitored every 10 min at Ex/Em = 490/520 nm during 8 hrs using a FLUOstar Optima microplate reader (BMG Labtech). In some experiments, the MMPs inhibitor GM6001 (Galardin/Ilomastat) (Santa Cruz Bio.) was used at 50 μM to pre-treat the fibroblasts CMs during 15 min. GM6001 was maintained at the same concentration during the 48 hrs treatment of T1 target cells with the fibroblast CMs or during the measurement of MMPs activity.

### MICA and MICB ELISA

0.1 × 10^6^ T1 target cells were treated in 12-w plates during 48 hrs with 500 μL DMEM/F12 medium supplemented with 5% FCS (ctrl) or with the CAFs CMs. The cell culture supernatants were then immediately frozen at −80°C. Soluble MICA and MICB concentration in these supernatants were independently measured by ELISA (Thermo Scientific) according to the manufacturer recommendations.

### Analysis of IFN-γ production by NK cells

T1 melanoma cell line, untreated or pre-treated during 48 hrs with the conditioned media of CAFs (CAF1-4) in the presence or absence of the MMPs inhibitor GM6001 (50 μM) were co-cultivated during 6 hrs with resting NK cells isolated from a healthy donor (NKd1) at the effector:target ratio of 3:1. Co-cultures were performed in the presence of 10 μg/mL Brefeldin-A (Sigma) to inhibit IFN-γ secretion. After 6 hrs, cells were harvested, washed with PBS and stained on ice with A488-conjugated mouse anti-CD56 mAb (clone B159; BD Biosciences). Cells were then washed 3 times with PBS, permeabilized with CytoFix/CytoPerm solution (BD Biosciences) and stained with APC-conjugated mouse anti-IFN-γ mAb (clone B27; BD Biosciences) before flow cytometry analysis of IFN-γ positive cells, gating on CD56^+^ NK cells.

### Detection of effector /target cell conjugates

Control or CMs pre-treated T1 and NK92 cells were respectively stained during 10 min with the lypophilic dyes DiO or DiD (Molecular Probes) according to the manufacturer's instructions and washed 3 times with warm culture medium. T1 target cells were then incubated with NK92 effector cells at the effector:target ratio of 3:1 in Ca^2+^ free medium (HBSS with 10 mM Hepes pH7.5, 0.4% BSA) during 30 min at 37°C to allow conjugate formation before flow cytometry analysis.

### Apoptosis assays

To assess PFN/GzmB-dependent apoptosis, T1 cells incubated for 2 hr at 37°C with buffer or sublytic PFN ± 50 nM hGzmB, were analyzed for caspase activation by flow cytometry using M30-FITC mAb staining (M30 CytoDEATH, Roche) to detect an effector caspase-cleavage product of cytokeratin 18, as previously described [37, 38]. Native rat PFN was purified from RNK16 cells and native hGzmB was purified from YT-Indy cells as described [64].

### Statistical analysis

Data are expressed as mean ± standard deviation (s.d.). *P* values were determined by unpaired two-tailed student's *t* tests.

## SUPPLEMENTARY MATERIALS FIGURES



## Abbreviations

CAF: Cancer-Associated Fibroblast
CM: Conditioned Medium
Gzm: Granzyme
IFN-γ: Interferon gamma
MICA/B: MHC class I polypeptide-related protein A and B
MMP: Matrix metalloproteinase
NF: Normal Fibroblast
NK: Natural Killer cell
NKG2D: Natural Killer Group 2 member D
PFN: Perforin
ULBP: HCMV UL16-binding proteins.
