# Supplementary material for: Melanoma-associated fibroblasts decrease tumor cell susceptibility to NK cell-mediated killing through matrix-metalloproteinases secretion
Source: Oncotarget. 2017 Feb 20;8(12):19780–94. doi: 10.18632/oncotarget.15540 (PMC5386721; doi:10.18632/oncotarget.15540)
Supplement: Supplementary file 1 [file oncotarget-08-19780-s001.pdf]

Melanoma-associated fibroblasts decrease tumor cell susceptibility to NK cell-mediated killing through matrix-metalloproteinases secretion

Supplementary Materials

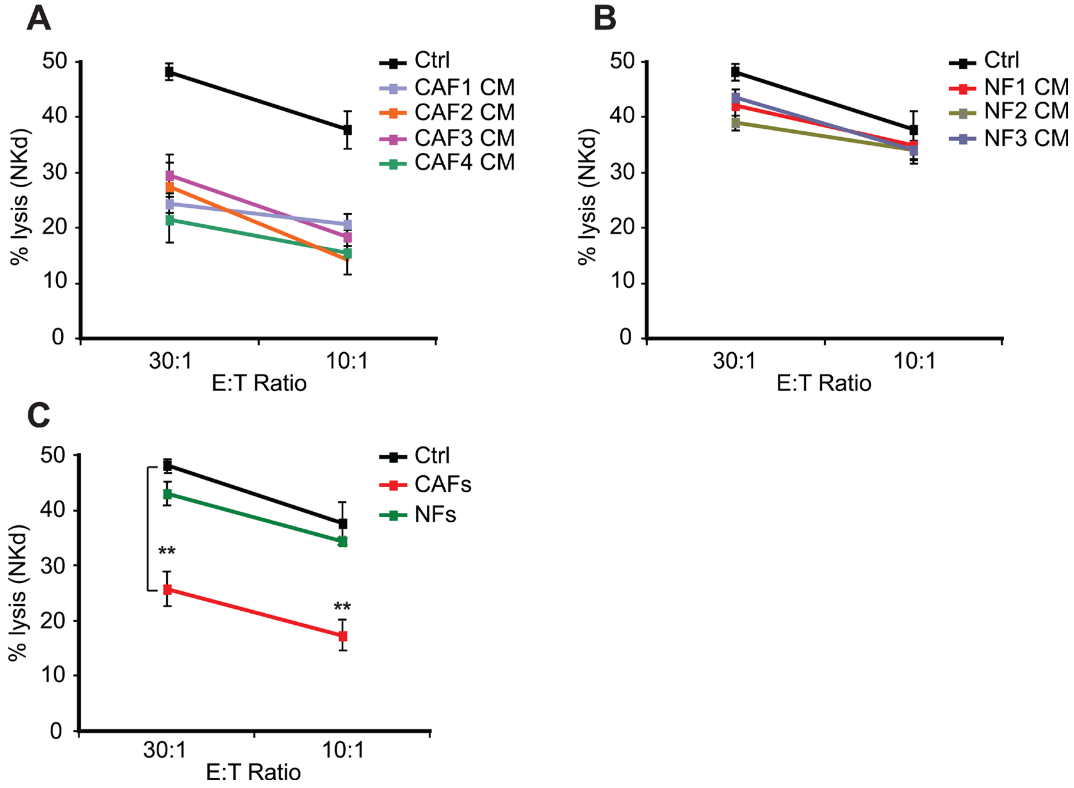

Supplementary Figure 1: Conditioned media from melanoma-associated fibroblasts decrease the susceptibility of WM17-16 melanoma tumor cells to NK-mediated killing. The lysis of the WM17-16 melanoma cell line, untreated (Ctrl) or pre-treated during 48 hrs with the conditioned media (CM) of CAFs (CAF1-4) (**A**) or normal skin fibroblasts (NF1-3) (**B**) by NK cells isolated from a healthy donor (NKd1) was evaluated by ^{51}Cr release assays at different effector:target (E:T) ratios. Data are the mean ± s.d. from three independent experiments. Experiments in (A–B) were performed at the same time but separated in two different panels. (**C**) represents the mean ± s.d. of all the NKd-mediated lysis experiments from (A-B) using the CAFs or NFs pre-treatments of the WM17-16 tumor cell line. *P* values (C) were determined by unpaired two-tailed student's *t*-test comparing the control and CAFs CMs pre-treatments. (**p < 0.0001).

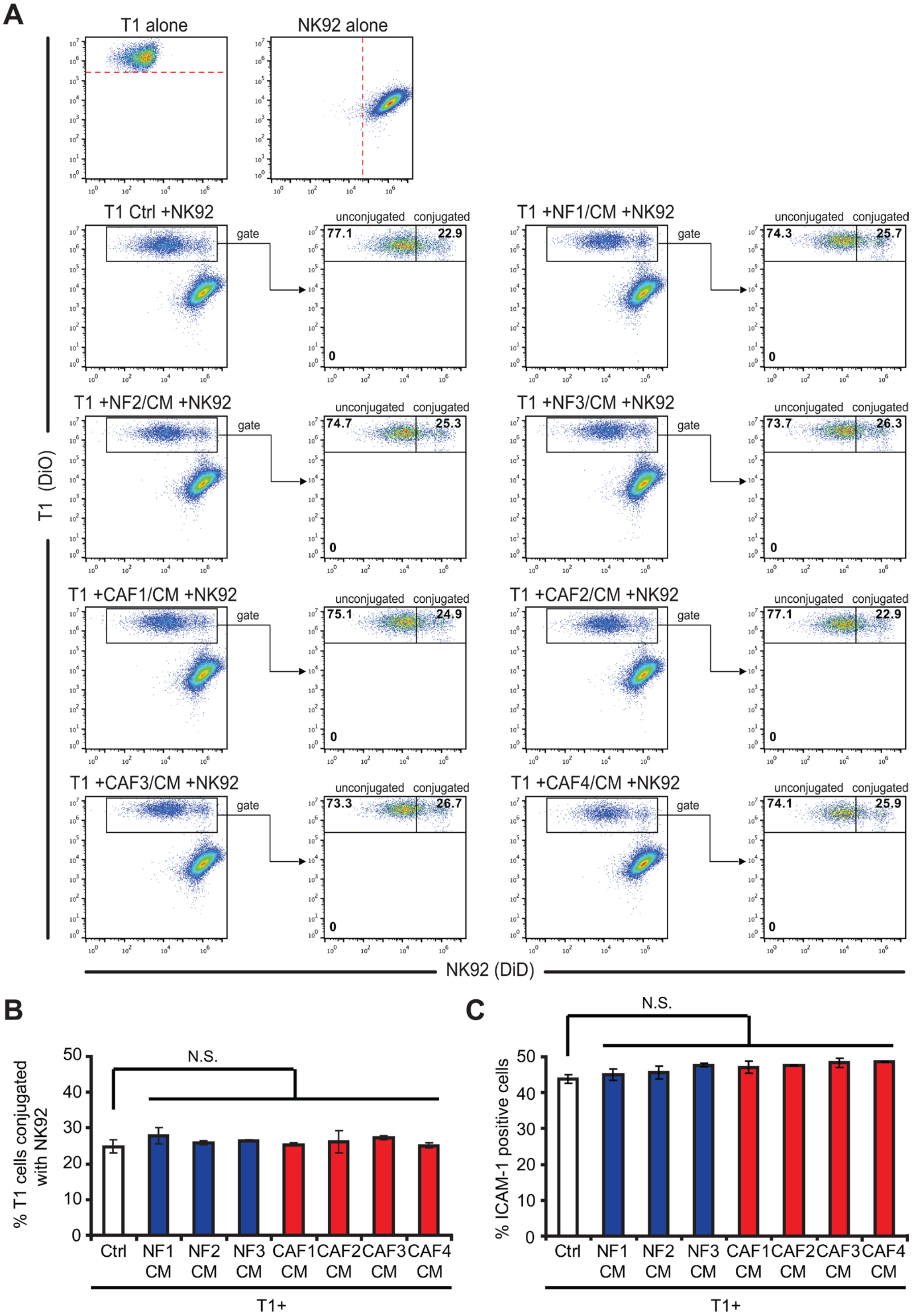

Supplementary Figure 2: Conditioned media from melanoma-associated fibroblasts do not alter the formation of conjugates between T1 target cells and NK92 effector cells. (**A–B**) CAF1-4 or NF1-3 CMs pre-treated T1 or control cells, stained with the lypophilic dyes DiO were incubated with NK92 effector cells, stained with the lypophilic dyes DiD, at the effector:target ratio of 3:1 during 30 min before measuring immune conjugates formation by flow cytometry. Isolated T1 and NK92 were used as control to identify the two separated populations. The percentage of T1 cells conjugated with NK92 cells was calculated by gating on T1 cells and on measuring the percentage of DiO/DiD double positive population representing the immune conjugates. Representative flow cytometry dot plots (A) and mean ± s.d. of percentage T1 cells conjugated with NK92 cells (B) from three independent experiments are shown. (**C**) ICAM-1 expression was evaluated by flow cytometry at the surface of control, CAF1-4 or NF1-3 CMs pre-treated T1 tumor cells. Data are the mean ± s.d. of percentage ICAM-1 positive T1 cells from three independent experiments. *P* values (B–C) were determined by unpaired two-tailed student's *t*-test. (N.S: non significant).

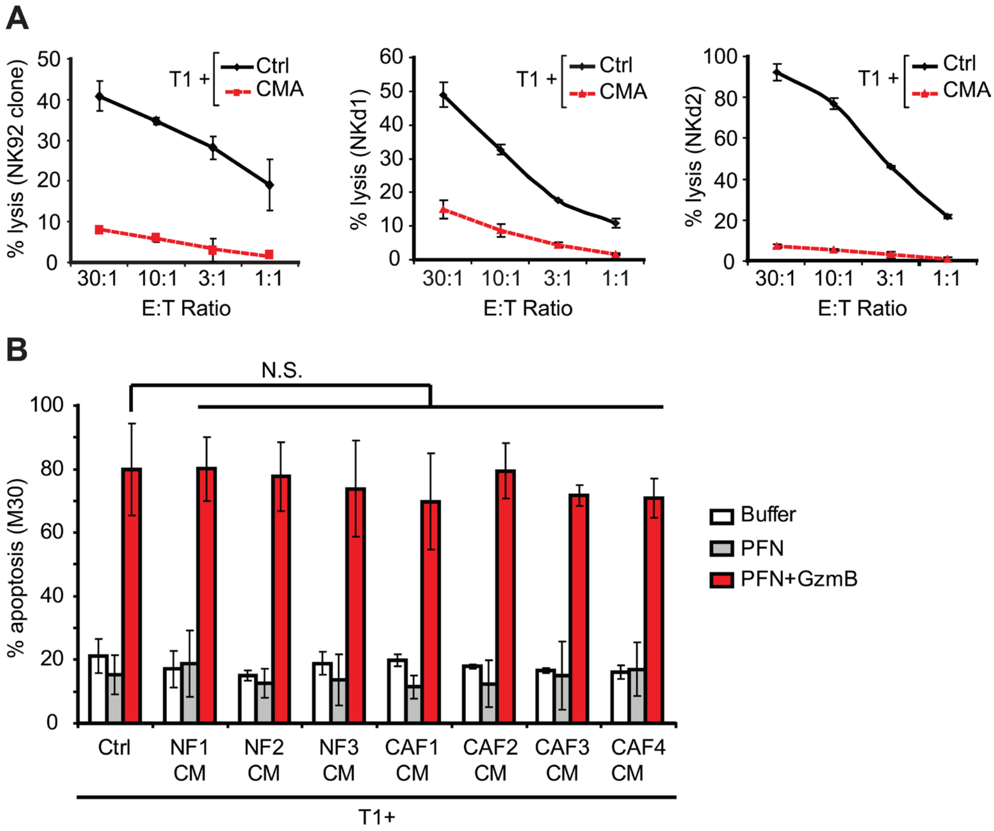

Supplementary Figure 3: NK-mediated lysis of T1 tumor cells is mainly dependent on the Perforin/Granzymes pathway but conditioned media from melanoma-associated fibroblasts do not alter the susceptibility of T1 cells to Perforin/Granzyme B-mediated apoptosis. (**A**) T1 tumor cells are killed by NK cells using the Perforin/Granzymes (PFN/Gzm) pathway. The lysis of the T1 melanoma cell line by NK cells (NK92 or NK cells isolated from two healthy donors (NKd1 and NKd2)) was evaluated by ^{51}Cr release assays at different effector:target (E:T) ratios. Pre-incubation of NK cells with 100 nM concanamycin A (CMA) (concentration of CMA during lysis: 50 nM), which inhibits calcium-dependent exocytosis of cytotoxic granules, was used as an inhibitor of PFN/Gzm-dependent lysis. Data are the mean ± s.d. from two independent experiments performed in triplicate. (**B**) Conditioned media from CAFs do not reduce the susceptibility of T1 cells to PFN/GzmB-mediated apoptosis. T1 cells untreated (Ctrl) or pre-treated during 48 hrs with the conditioned media (CM) of CAFs (CAF1-4) or normal skin fibroblasts (NF1-3) were treated with PFN ± 50 nM hGzmB during 2 hrs. Apoptosis was measured by flow cytometry using M30 mAb staining (which recognizes a cytokeratin-18 epitope, revealed after effector caspase cleavage). Mean ± s.d. of percentage M30 positive cells from three independent experiments are shown. *P* values were determined by unpaired two-tailed student's *t*-test. (N.S: non significant).

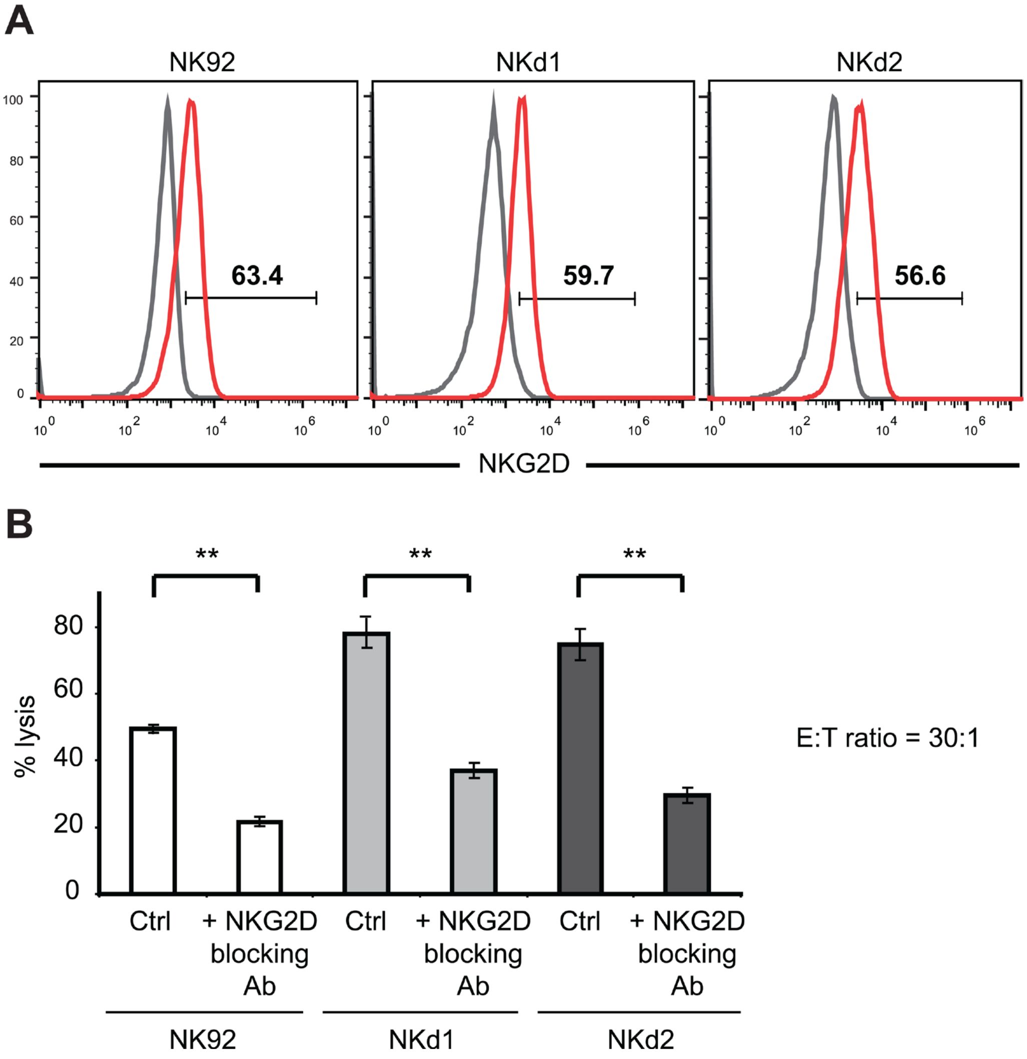

Supplementary Figure 4: NK-mediated lysis of T1 tumor cells is dependent on the NKG2D pathway. (**A**) NK92 and NK cells isolated from two healthy donors (NKd1 and NKd2) express the NKG2D activating receptor. Representative flow cytometry histograms from two independent experiments are shown. Gray lines represent the isotype controls. (**B**) Blocking NKG2D reduces T1 susceptibility to NK cell-mediated lysis. NK92, NKd1 or NKd2 effector cells were pre-incubated with 0.5 µg/mL anti-NKG2D blocking mAb (which was maintained at the same concentration during lysis) before incubation with T1 target cells. T1 lysis was evaluated by ^{51}Cr release assays at the effector:target (E:T) ratios of 30:1. Data are the mean ± s.d. from two independent experiments performed in triplicate. *P* values were determined by unpaired two-tailed student's *t*-test. (***p* < 0.001)
